# Clustering of sebaceous gland carcinoma, papillary thyroid carcinoma and breast cancer in a woman as a new cancer susceptibility disorder: a case report

**DOI:** 10.4076/1752-1947-3-6905

**Published:** 2009-07-16

**Authors:** Brian D Newman, Joseph F Maher, Jose S Subauste, Gabriel I Uwaifo, Steven A Bigler, Christian A Koch

**Affiliations:** 1 Department of Medicine, Division of Endocrinology, University of Mississippi Medical Center, Jackson, MS 39216, USA; 2 Department of Medicine, Mayo Clinic, 200 1st St SW, Rochester, MN 55905, USA; 3 Departments of Medicine and Pediatrics, Center for Human Genetics, University of Texas Southwestern Medical Center, Dallas, TX 75390, USA; 4 GV (Sonny) Montgomery VA Medical Center, 1500 East Woodrow Wilson Drive, Jackson, MS 39216, USA; 5 Department of Pathology, University of Mississippi Medical Center, Jackson, MS 39216, USA

## Abstract

**Introduction:**

Multiple distinct tumors arising in a single individual or within members of a family raise the suspicion of a genetic susceptibility disorder.

**Case presentation:**

We present the case of a 52-year-old Caucasian woman diagnosed with sebaceous gland carcinoma of the eyelid, followed several years later with subsequent diagnoses of breast cancer and papillary carcinoma of the thyroid. Although the patient was also exposed to radiation from a pipe used in the oil field industry, the constellation of neoplasms in this patient suggests the manifestation of a known hereditary susceptibility cancer syndrome. However, testing for the most likely candidates such as Muir-Torre and Cowden syndrome proved negative.

**Conclusion:**

We propose that our patient's clustering of neoplasms either represents a novel cancer susceptibility disorder, of which sebaceous gland carcinoma is a characteristic feature, or is a variant of the Muir-Torre syndrome.

## Introduction

Multiple distinct tumors arising in a single individual or within members of a family raise the suspicion of a genetic susceptibility disorder [1,2]. Tumor suppressor genes, such as *PTEN* in Cowden syndrome and *BRCA1/2* in breast cancer, function by eliciting apoptosis and G1 cycle arrest. However, expression and tissue-specific splicing may lead to the differential expression of splice variants (SVs) with subsequent downstream signaling consequences. SVs resulting from alterations in the splicing of cancer-related genes could represent novel cases of familiar syndromes that do not reveal classic mutations.

The diagnosis of sebaceous gland carcinoma can represent a marker of an associated heritable disorder, and some authors recommend patients be evaluated for other visceral malignancies [3]. Additionally, prompt diagnosis enables routine surveillance of occult cancers, identification of low-grade tumors that would be more responsive to treatment, and identification of family members at risk for developing cancer.

## Case presentation

**Figure 1:**
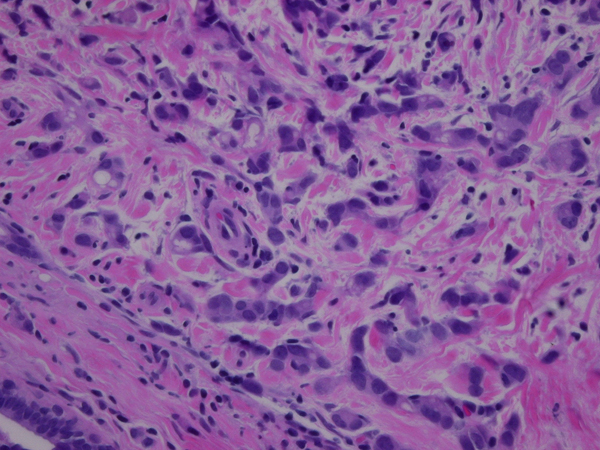
**Infiltrating ductal carcinoma of the right breast, Grade 2**. H & E stain, original magnification 400x.

A 52-year-old Caucasian woman from Mississippi with a history of sebaceous carcinoma of the left lower eyelid, breast cancer of the right breast (ER+/PR+/HER2+), and papillary thyroid cancer treated by total thyroidectomy and radioactive iodine ablation (49.5 mCi) was referred to our clinic for follow-up evaluation. Sebaceous gland carcinoma of the eye was diagnosed when the patient was 43-years-old and surgically treated at a hospital in Pennsylvania. She was then diagnosed with multifocal ductal carcinoma in situ of the right breast (Figure 1) in 2004 (T2N0M0), for which testing showed to be ER/PR+ as well as HER2+.

Following simple unilateral mastectomy, she initially underwent eight cycles of chemotherapy and tamoxifen treatment, but stopped taking tamoxifen secondary to fears of developing endometrial cancer in 2006. At that time, her hormone levels were checked revealing a low estradiol level (13.9 pg/ml) and an appropriately elevated follicle-stimulating hormone (FSH) (63 mIU/ml), indicative of menopause.

A mammogram from 2008 was unremarkable for recurrent cancer and she denied any vaginal bleeding. In 2005, a papillary thyroid carcinoma (size: 0.6 cm) was diagnosed after she had been found to have a thyroid nodule on routine ultrasound for which a right lobectomy was initially performed, but later followed by a total thyroidectomy in light of her multiple prior cancers.

She subsequently received ablative therapy with 49.5 mCi of ^131^Iodine in 2006. Since that time, she has remained asymptomatic and is taking daily calcium tablets as well as 150 mcg of thyroxine. Serum thyroglobulin levels had been undetectable (<0.1 ng/ml) while thyroglobulin antibodies were measured at 12 IU/ml (normal, <4.0), and thyroid stimulating hormone (TSH) at 0.006 mcU/ml (0.23 to 4.0 mcU/ml). Sonographic examinations of her neck had been non-suspicious for recurrent cancer. Given her postmenopausal and iatrogenically-induced hyperthyroid state, she underwent a bone mineral density study that revealed osteopenia at the spine and the femur (T-score was minus 1.1). Her 25-hydroxy vitamin D level was normal at 41 ng/ml.

The patient's family history was positive for breast cancer in her mother and maternal aunt. One of the patient's paternal first degree cousins suffered from an inoperable brain tumor, and a male and a female paternal second degree cousin had breast cancer. However, her family history was negative for colon cancer, endometrial or ovarian cancers, thyroid cancer, or any other cancers.

The patient recollected that her husband, who died of colon cancer, had been gainfully employed welding and cutting pipe that was previously used in the oil field industry. She further remembered being proximate to the pipe on multiple occasions, when she was helping her husband cut and weld sections, with resultant fume inhalation. After his death, she was unable to sell the pipe because it was determined to be too radioactive from its prior use in the oil field to be sold on the open market. The determination of radioactivity was made using a Geiger counter; however, the type of radiation was not determined.

Based on her personal and family history of multiple cancers, the patient underwent genetic testing for Cowden syndrome (*PTEN* gene testing for mutations in exons 1 to 9 was negative), screening colonoscopy (negative) to exclude Muir-Torre syndrome, hereditary nonpolyposis colorectal cancer (HNPCC) or a variant of HNPCC, and immunohistochemistry (IHC)/microsatellite instability (MSI) testing on her breast cancer specimen for HNPCC (IHC: normal pattern for hMLH1, hMSH2, hMSH6, hPMS2; MSI: BAT25, BAT26, BAT40, BAT34c mononucleotide repeats, D17s250, D5s346, D18s55, D10s197, MycL, ACTC dinucleotide). We could not rule out a *PTEN* promoter mutation, because this was not tested. Given a Manchester score of 5-7 for BRCA2 with a less than 5% chance of finding a BRCA2 mutation, mutation analysis in BRCA1 or BRCA2 was not pursued.

## Discussion

Sebaceous gland carcinoma is a rare neoplasm arising from the Meibomian glands, Zeis glands, or sebaceous glands of the caruncle and eyebrow, and represents 1-6% of all eyelid malignancies [4,5]. The median age at diagnosis is 72 years, with most diagnoses prior to 30 years of age revealing a history of radiation exposure [4,6]. Moreover, among individuals diagnosed with sebaceous gland carcinoma, the incidence of one or more primary visceral malignancies has been noted to be as high as 42% [7].

In their report, Finan and Connolly [7], listed thyroid adenomas, uterine fibroids, and benign renal cysts as less common findings. Papillary thyroid carcinoma (PTC) is the most common thyroid malignancy worldwide, comprising 50% to 70% of differentiated follicular cell thyroid carcinomas [8]-[10]. PTC is strongly associated with prior irradiation to the head and neck, likely resulting in chromosome breakage and rearrangement with fusion of the *RET* tyrosine kinase domain to various breakpoint sites, clearly demonstrated in post-Chernobyl thyroid tumors [11,12]. Familial clustering of PTC is well recognized and family studies have revealed autosomal dominant transmission [13]. The high incidence of PTC in patients with adenomatous polyposis and Cowden syndrome (the multiple hamartoma syndrome) suggests a shared set of susceptibility genes. The genetic differential diagnosis for papillary thyroid carcinoma is shown in Table 1.

**Table 1:** Genetic Differential Diagnosis of Thyroid Cancer Associated Syndromes

Histology	Syndrome Association	Gene (if known)
Medullary	MEN2	*RET*
Follicular	Cowden Syndrome	*PTEN*
	Werner Syndrome	*WRN*
Papillary	Familial Adenomatous Polyposis	*APC*
	Cowden Syndrome	*PTEN*
	Carney Complex	*PRKAR1A*
	Familial Nonmedullary Thyroid Cancer Syndrome	
	Familial Papillary Thyroid Carcinoma	

Adapted from: Weber F, Eng C. Update on the Molecular Diagnosis of Endocrine Tumors: Towardâ€“omics-Based Personalized Healthcare? *J Clin Endocrinol Metab* 2008, **93**(4):1097-1104.

The strong family history of breast neoplasms in our patient further suggests an increased susceptibility to cancer. Breast cancer is the most common cancer in women and has been associated with a number of specific genetic mutations, namely BRCA1/2, which accounts for approximately 5% of reported cases [14]. Less frequently implicated in breast cancer are *PTEN* in Cowden syndrome, MLH1 and MSH2 in Muir-Torre, and STK11 in Peutz-Jeghers syndrome [15]-[17].

Our patient presented with multiple malignant neoplasms including sebaceous gland carcinoma, papillary thyroid carcinoma, and breast cancer. Her mother who died at age 73 and maternal aunt who died at age 67 with a diagnosis of breast at around age 50, were both diagnosed with breast cancer, but both did not suffer any of the other neoplasms that were present in our patient. The clustering of visceral malignancies with sebaceous carcinoma in our patient is a unique occurrence and strongly suggests the likelihood of an underlying cancer susceptibility disorder, perhaps an under-recognized manifestation or variant of the Muir-Torre syndrome.

We considered whether these observations might be explained in the context of a known genetic cancer susceptibility disorder and felt that Muir-Torre syndrome (MTS), Cowden syndrome (CS), and Carney complex (CRC) were the most likely candidates, although none completely accounts for the pattern of neoplasms in this patient.

Muir-Torre syndrome (MTS) is an autosomal dominant disorder characterized by sebaceous gland carcinoma and one or more internal visceral malignancies (Table 2). The etiology of this disorder is thought to result from a mutation in the mismatch DNA repair genes *MSH-2* and* MLH-1*, supported by the finding of microsatellite instability in tumors of some patients [18,19]. However, one study shows that 31% of patients with MTS tumors exhibited no microsatellite instability, which suggests the existence of at least two variants of MTS with different molecular mechanisms [20]. MTS has also been described in the setting of MYH-related attenuated polyposis, resulting from a mutation in the MYH gene that caused aberrations in base excision repair. However, its role in the development of cutaneous sebaceous carcinogenesis is unclear [21]. Patients with MTS often have colonic polyps and adenomas, but neither is necessary to make the diagnosis. The most commonly identified neoplasms in MTS are shown in Table 3. Conventional testing on the breast cancer specimen of our patient was indicative of normal DNA mismatch repair function within the tumor. We did not perform mutation analysis of *MSH-2* and* MLH-1.*

**Table 2:** Criteria for Diagnosis of Muirr-Torre Syndrome (MTS)

At least one:
Sebaceous Carcinoma
Sebaceous Epithelioma
Sebaceous Adenoma
Keratoacanthoma with Sebaceous differentiation
And:
1 or more visceral malignancies
**OR:**
All of the following:
1) Family history of MTS
2) Multiple visceral malignancies
3) Multiple Keratoacanthomas

Adapted from: Weinstein et al. Muirr-Torre syndrome: a case of this uncommon entity. *Int J Dermatol* 2006, **45**:311-313.

**Table 3:** Incidence of Internal Malignancies in MTS

Site	Incidence (%)
Colon	49%
Genitourinary	21%
Breast	11%
Hematologic	9%
Head/Neck	4%
Small Intestine	2%
Other	4%

Modified from: Weinstein et al. Muirr-Torre syndrome: a case of this uncommon entity. *Int J Dermatol* 2006, **45**:311-313.

Cowden syndrome (CS) is an autosomal dominant disorder distinguished by pathognomonic mucocutaneous lesions such as facial trichilemmomas, acral keratoses and papillomatosis; hamartomatous polyps, and internal visceral malignancies including breast and thyroid cancer. The causative mutation in this syndrome involves the *PTEN* tumor suppressor gene, resulting in an unregulated progression through the G1 phase of the cell cycle.

Our patient had breast and thyroid cancer, but her lack of hamartomatous polyps and the presence of a sebaceous carcinoma cannot be readily explained in the framework of this syndrome. Furthermore, there have been no reports of sebaceous carcinoma in a patient with CS. PTC is a cancer strongly linked with radiation exposure to the head and neck and the proximity of the sebaceous carcinoma suggests that both could have resulted from a single field of exposure, in this case the radioactive piping that the patient was exposed to. Commercial testing for germline mutations in *PTEN* failed to show any in exons 1-9. However, a mutation is identified in only 80% of patients who meet clinical criteria [22,23]. On the other hand, recent data suggest that germline sequence variants in various tumor suppressor and/or oncogenes may cooperatively promote tumorigenesis of various tumor types including thyroid cancer [24]-[32].

Meanwhile, Carney complex is an autosomal dominant disorder that leads to endocrine gland tumors and/or hyperfunctioning commonly involving the pituitary, adrenal, and thyroid glands, as well as myxomas (skin, heart, and breast) and lentiginosis in select areas of the skin. The causative mutation involves the regulatory subunit of protein kinase receptor 1A (*PRKAR1A*), which results in nonsense-mediated decay of the transcript and altered protein kinase A signaling [13]. Again, the presence of breast and thyroid neoplasms is suspect, but sebaceous gland carcinoma has not been described in the setting of Carney complex. We did not test for a germline mutation of the *PRKAR1A* gene, which is detectable in 50-65% of cases.

The field of genomics has provided new insight on the role that splicing and other mRNA processing mechanisms serve in the regulation of gene function. Numerous examples of alterations in splicing and differential expression of SVs and their role in various sporadic cancers have been reported [33]-[37]. Many of the genes involved in cancer susceptibility syndromes (*PTEN*, *APC*, MSH-2) are ubiquitously expressed and tissue-specific splicing may lead to differential expression of SVs, which may suggest different roles for different SVs in different tissues. In fact, one study identified SVs of *PTEN* that were expressed differentially in heritable cancer, sporadic cancer, and controls. It is believed that the nonlinear, tissue-specific expression of these SVs exert varying effects at the functional or regulatory level [38].

The implication of such research becomes especially important when considering that similar gene regulation and inactivation occurs in the inherited cancer syndromes, even in the absence of identifiable gene mutations. This may in part explain the relatively low sensitivity of modern laboratory techniques that rely heavily on isolating specific mutation sequences. For instance, abnormalities in the promoter region of a gene could be missed or deletions of one or more exons on one allele.

Additionally, measurement of mRNA content in peripheral blood may aid diagnosis as well as provide novel biomarkers for the identification of certain types of cancer, possibly circumventing the need for traditionally invasive techniques such as FNA, for example, in thyroid cancer (13). Furthermore, such discoveries could pave the way for targeting anti-apoptotic SVs to lower the apoptotic threshold of a tumor cell, thereby increasing the efficacy of chemotherapy drugs. Despite the emerging evidence linking mRNA level regulation and carcinogenesis, there is limited information regarding its role in many of the well-characterized familial cancer syndromes. Very few SVs have been identified and, unfortunately, many of those that have been identified were not attributed with any functional significance [38].

## Conclusions

Our patient, who had an unusual clustering of sebaceous gland carcinoma and internal malignancies, represents a unique clinical case. The most likely explanation would be that of either a single, unifying genetic cause resulting from a still undiscovered germline mutation, or cooperative tumorigenesis by germline sequence variants in various genes that play a role in tumor development. The history of radiation exposure was arguably the environmental catalyst for malignant transformation in this patient with an underlying genetic susceptibility. In the future, advancements in the field of genomics may allow further elucidation of the role of SVs in cancer susceptibility syndromes lacking identifiable genetic mutations.

We speculate that this patient's clustering of neoplasms represents a novel cancer susceptibility disorder, of which sebaceous gland carcinoma is a characteristic feature. This hypothesis cannot be tested on the basis of a single case report; therefore, we await further contributions from other clinical investigators.

## Consent

Written informed consent was obtained from the patient for publication of this case report and any accompanying images. A copy of the written consent is available for review by the Editor-in-Chief of this journal.

## Competing interests

The authors declare that they have no competing interests.

## Authors' contributions

BN and CK drafted the manuscript. JM initiated genetic testing. JM, JS, and GU provided valuable medical input. SB made photographs of important histological slides. All authors read and approved the final manuscript.
